# Identification and analysis of genes associated with head and neck squamous cell carcinoma by integrated bioinformatics methods

**DOI:** 10.1002/mgg3.857

**Published:** 2019-07-15

**Authors:** Yu Jin, Ya Yang

**Affiliations:** ^1^ Department of General Dentistry Ninth People's Hospital, Shanghai Jiao Tong University School of Medicine Shanghai PR China; ^2^ Shanghai Key Laboratory of Stomatology and Shanghai Research Institute of Stomatology National Clinical Research Center of Stomatology Shanghai PR China

**Keywords:** biomarker, GEO, head and neck squamous cell carcinoma, prognosis, TCGA

## Abstract

**Background:**

Head and neck squamous cell carcinoma (HNSCC) is one of the most common cancers worldwide, exhibiting high morbidity and mortality. The prognosis of HNSCC patients has remained poor, though considerable efforts have been made to improve the treatment of this cancer. Therefore, identifying significant differentially expressed genes (DEGs) involved in HNSCC progression and exploiting them as novel biomarkers or potential therapeutic targets for HNSCC is highly valuable.

**Methods:**

Overlapping differentially expressed genes (DEGs) were screened out from three independent gene expression omnibus (GEO) datasets and subjected to GO and kyoto encyclopedia of genes and genomes pathway enrichment analyses. The protein–protein interactions network of DEGs was constructed in the STRING database, and the top ten hub genes were selected using cytoHubba. The relative expression of hub genes was detected in GEPIA, Oncomine, and human protein atlas (HPA) databases. Furthermore, the relationship of hub genes with the overall survival and disease‐free survival in HNSCC patients was investigated using the cancer genome atlas data.

**Results:**

The top ten hub genes (SPP1, POSTN, COL1A2, FN1, IGFBP3, APP, MMP3, MMP13, CXCL8, and CXCL12) could be utilized as potential diagnostic indicators for HNSCC. The relative levels of FN1, APP, SPP1, and POSTN could be associated with the prognosis of HNSCC patients. The mRNA expression of APP and COL1A2 was validated in HNSCC samples.

**Conclusion:**

This study identified effective and reliable molecular biomarkers for diagnosis and prognosis by integrated bioinformatics analysis, suggesting novel and essential therapeutic targets for HNSCC.

## 1 INTRODUCTION

Head and neck squamous cell carcinoma (HNSCC) is ranked as the sixth most common cancer worldwide with approximately 550,000 new cases and 300,000 deaths each year (2016, & Jemal, 2^2016^). Although considerable efforts have been devoted to the improvement of traditional treatment methods including surgery, radiotherapy and chemotherapy, the overall 5‐year survival rate of HNSCC patients has remained unchanged over the past several decades (Miller et al., 2016). Late diagnosis, lymph node metastasis, and recurrences are the main reasons for the poor prognosis of HNSCC patients, leading to a high mortality rate of HNSCC (Duprez et al., 2017). Thus, investigating reliable and effective molecular markers and understanding essential genes involved in the biological process of HNSCC is urgently needed.

The Gene Expression Omnibus (GEO) database is a free online database that stores a multiple of high‐throughput microarray, chips, and next‐generation sequence functional genomic data sets (Clough & Barrett, 2016). The GEO database could be utilized to screen out differentially expressed genes (DEGs), to explore molecular signals and correlations, and to analyze gene regulatory networks. However, due to high expenses and limited sample tissues, the analysis results of individual experiments may be biased and unreliable. Therefore, integrated analyses of multiple datasets may improve the accuracy and reliability of the analysis and hence produce a more comprehensive and well‐rounded discovery of DEGs in a variety of cancers. For example, the usage of two GEO datasets provided a novel understanding of the mechanism underlying breast cancer metastasis to the brain and provided potential therapeutic targets (Tang, Zhao, Zhang, Wang, & Wang, 2019). Additionally, by analyzing the original data (GSE13911, GSE19826, GSE79973 and GSE29272) from the GEO database, the most significant genes and pathways associated with gastric cancer were identified, providing potential therapeutic targets to improve the clinical effects in patients with gastric cancer (Fei et al., 2018). What is more, dysregulated genes identified from four independent GEO datasets were found to be closely associated with hepatocellular carcinoma progression and may be exploited as potential biomarkers for diagnosis and prognosis (Yin et al., 2016).

In this study, original data from microarray analyses conducted on HNSCC samples were downloaded from the GEO database, and integrated analysis was implemented. A total of 115 overlapping DEGs were screened out by the intersection of three independent datasets. Then, GO/kyoto encyclopedia of genes and genomes (KEGG) pathway analysis and protein–protein interactions (PPI) were performed to evaluate the underlying molecular mechanisms involved in carcinogenesis and tumor progression. The hub genes that may play essential roles in HNSCC were identified using cytoHubba tool kits in the Cytoscape software. The relative expression level of hub genes and their relationship with HNSCC patient survival were validated in multiple online databases.

## 2 MATERIALS AND METHODS

### 2.1 Ethical compliance

This study was approved by the Review Board of the Medical Ethics Committee of the Ninth People's Hospital, Shanghai Jiao Tong University School of Medicine. A total of 32 pairs of HNSCC tissues and matched adjacent normal tissues were obtained from the patients who were diagnosed with primary HNSCC and underwent initial surgery at the Department of Oral Maxillofacial‐Head and Neck Oncology, Ninth People's Hospital, Shanghai Jiao Tong University School of Medicine (Shanghai, China). Informed consent and approval were obtained from all patients. All of the tissue samples were frozen in liquid nitrogen for further experiments. In this study, none of these HNSCC patients received any preoperative cancer treatment and were histologically diagnosed as HNSCC after operation.

### 2.2 Microarrays and bioinformatics analysis

The original CEL files of three independent GEO (Clough & Barrett, 2016) datasets (GSE13601, GSE31056, GSE30784) were downloaded and analyzed using R language. We utilized the affy package to perform the background correction and data normalization, including conversion of raw data formats, imputation of missing values and background correction. Then, the samples were subjected to differential expression analysis using the Limma package. *p* < .01 and |log(FC)| >1 were set as the threshold, and the genes that met the criteria were screened out as DEGs. The intersection of DEGs from three datasets was performed using the VennDiagram package in R language.

### 2.3 Gene ontology and KEGG pathway analysis

KOBAS v3.0 (http://kobas.cbi.pku.edu.cn) is a web server for gene/protein functional annotation and functional gene set enrichment (Wu, Mao, Cai, Luo, & Wei, 2006). The overlapping DEGs from three GEO datasets were subjected to Gene Ontology and KEGG pathway analysis by this online tool. *p* < .05 was considered as statistically significant.

### 2.4 Identification of top modules and hub genes in a PPI network

Exploring the functional interactions between proteins is essential for understanding the molecular mechanisms of carcinoma. The Search Tool for the Retrieval of Interacting Genes (STRING v11.0) is an online tool (https://string-db.org/) designed to establish potential interactions among a good number of genes (von Mering et al., 2003). The overlapping DEGs were put into the software to construct the PPI network and were then visualized using Cytoscape software 3.7 (http://www.cytoscape.org; Shannon et al., 2003). In addition, the Molecular Complex Detection (MCODE) in Cytoscape software (Cytoscape v3.7.1) was applied to screen out top modules inside the PPI network with degree cut‐off = 2, node score cut‐off = 0.2, Max depth = 100 and k‐score = 2. CytoHubba, a plugin in the Cytoscape software, was adopted to calculate the degree of each protein node. In our study, the top ten genes were selected as hub genes.

### 2.5 Validation of relative mRNA expression levels of hub genes

To further validate the mRNA level of hub genes in HNSCC, we examined the relative expression of these genes in two databases, namely, Oncomine and GEPIA. GEPIA v1.0 (Gene Expression Profiling Interactive Analysis; http://gepia.cancer-pku.cn/), a web‐based tool based on the cancer genome atlas (TCGA) and GTEx data, could be utilized to conduct differential expression analysis, correlation analysis, patient survival analysis, similar gene detection and dimensionality reduction analysis (Tang et al., 2017). Oncomine v4.5 (https://www.oncomine.org/), an online platform providing cancer microarray datasets and data‐mining functions, could be applied to validate the expression of specific genes in multiple cancers, thereby facilitating the discovery of potential essential genes involved in tumor development and progression (Rhodes et al., 2004). In the current study, we detected the relative expression of ten hub genes in these two databases with a threshold of *p *˂ .05 and a fold change of 2.

### 2.6 Exploration of the protein levels of hub genes in the human protein atlas database

The human protein atlas (HPA v18.1) database (https://www.proteinatlas.org/) is an online free database that provides abundant transcriptome and proteome data on human normal or pathological tissues through RNA‐sequencing analysis and immunohistochemistry analysis (Uhlen et al., 2015). In this study, the protein expression and distribution of hub genes was investigated in HNSCC tissues and compared normal tissues in HPA.

### 2.7 Survival analysis of hub genes based on the TCGA database

UCSC Xena v1.0 (http://xenabrowser.net/) is an online database from which users could obtain functional genomic data sets to make correlations between genomic and/or phenotypic variables. In this study, we utilized this free online tool to detect whether the expression of hub genes was correlated with the survival of HNSCC patients from TCGA samples. Patients were grouped into a relatively high expression group and a low expression group according to the median, and *p* < .05 was considered as statistically significant.

### 2.8 RNA extraction and quantitative real‐time PCR

Total RNA from the clinical samples was extracted using Trizol reagent (Takara), and complementary cDNA was synthesized by using a PrimeScript™ RT reagent kit (Takara). Real‐time PCR was performed on an ABI StepOne real‐time PCR system (Life Technologies) by using a SYBR Premix Ex Taq Reagent Kit (Takara). The reaction conditions were as follows: 95°C for 5 min, 40 cycles of 5 s at 95°C, and 30 s at 60°C. All the primers were designed and synthesized by Sangon Biotech (Shanghai) and the detailed sequences for the primers are as follows. APP (forward primer: 5'‐CAAGCAGTGCAAGACCCATC‐3' and reverse primer: 5'‐AGAAGGGCATCACTTACAAACTC‐3'), COL1A2 (forward primer: 5'‐GAGCGGTAACAAGGGTGAGC‐3' and reverse primer: 5'‐CTTCCCCATTAGGGCCTCTC‐3'), and GAPDH (forward primer: 5'‐ACAACTTTGGTATCGTGGAAGG‐3' and reverse primer: 5'‐GCCATCACGCCACAGTTTC‐3'). GAPDH was used as the internal reference in this study. The relative expression level was calculated by adopting the 2^−ΔΔCt^ method and all experiments were repeated in triplicate.

### 2.9 Statistical analysis

All the statistical analysis in this study was performed using spss 21.0 software. Comparisons between the two groups were performed using Student's two‐tailed *t* test. Kaplan–Meier survival analysis was performed to compare HNSCC patient survival based on hub gene expression using log‐rank test. *p* < .05 was considered statistically significant.

## 3 RESULTS

### 3.1 Identification of DEGs from GEO datasets analysis

**Figure 1:**
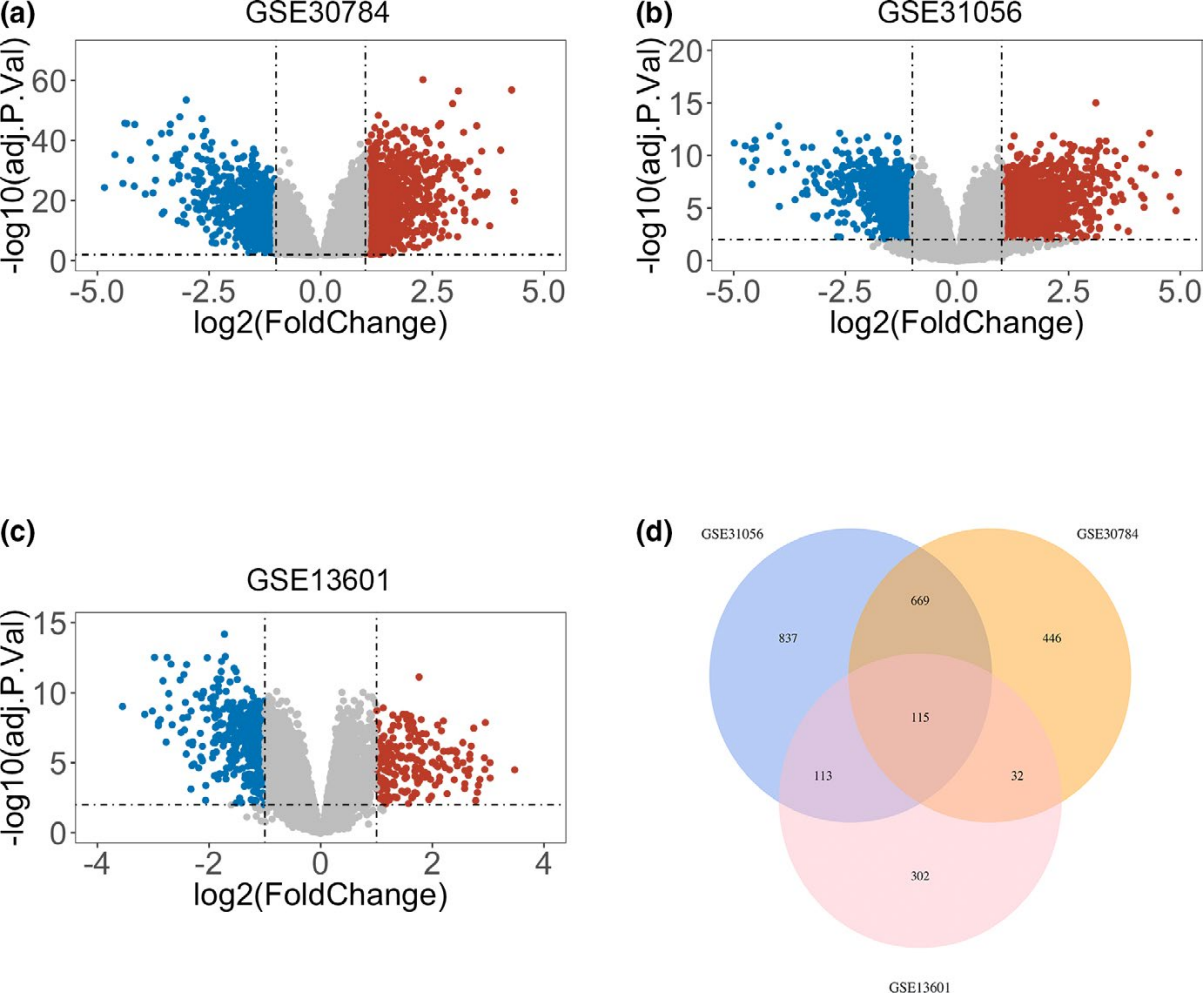
Identification of differentially expressed genes (DEGs) in three gene expression omnibus (GEO) datasets. (a) Volcano plot of DEGs in GSE30784. (b) Volcano plot of DEGs in GSE31056. (c) Volcano plot of DEGs in GSE13601. Red, blue and gray color represent relatively high, low and equal expression of genes in the corresponding group, respectively. *p* < .01 and |log(FC)| >1 were set as the threshold. (d) Venn diagram of overlapping DEGs from intersection of three independent GEO datasets

Raw data from three independent datasets (GSE31056, GSE30784, GSE13601) were downloaded from GEO and then subjected to differential expression analysis using R language. The detailed information about GEO datasets is summarized in Table 1. Genes screened from the criteria set as *p* < .01 and |log(FC)| >1 were plotted using R language to visualize the distribution of DEGs between HNSCC and compared normal controls from three studies (Figure 1a‐c). Red or blue dots represent significantly upregulated or downregulated genes, respectively. Afterwards, common DEGs from all three datasets were identified using a Venn diagram in R language (Figure 1d).

**Table 1:** Details of the gene expression omnibus (GEO) datasets

Reference	Sample	GEO	Platform	Normal	Tumor
Estilo et al. (2009)	Oral	GSE13601	GPL8300	27	31
Reis et al. (2011)	Oral	GSE31056	GPL10526	24	23
Chen et al. (2008)	Oral	GSE30784	GPL570	45	167

### 3.2 GO analysis and KEGG analysis of the overlapping DEGs

**Figure 2:**
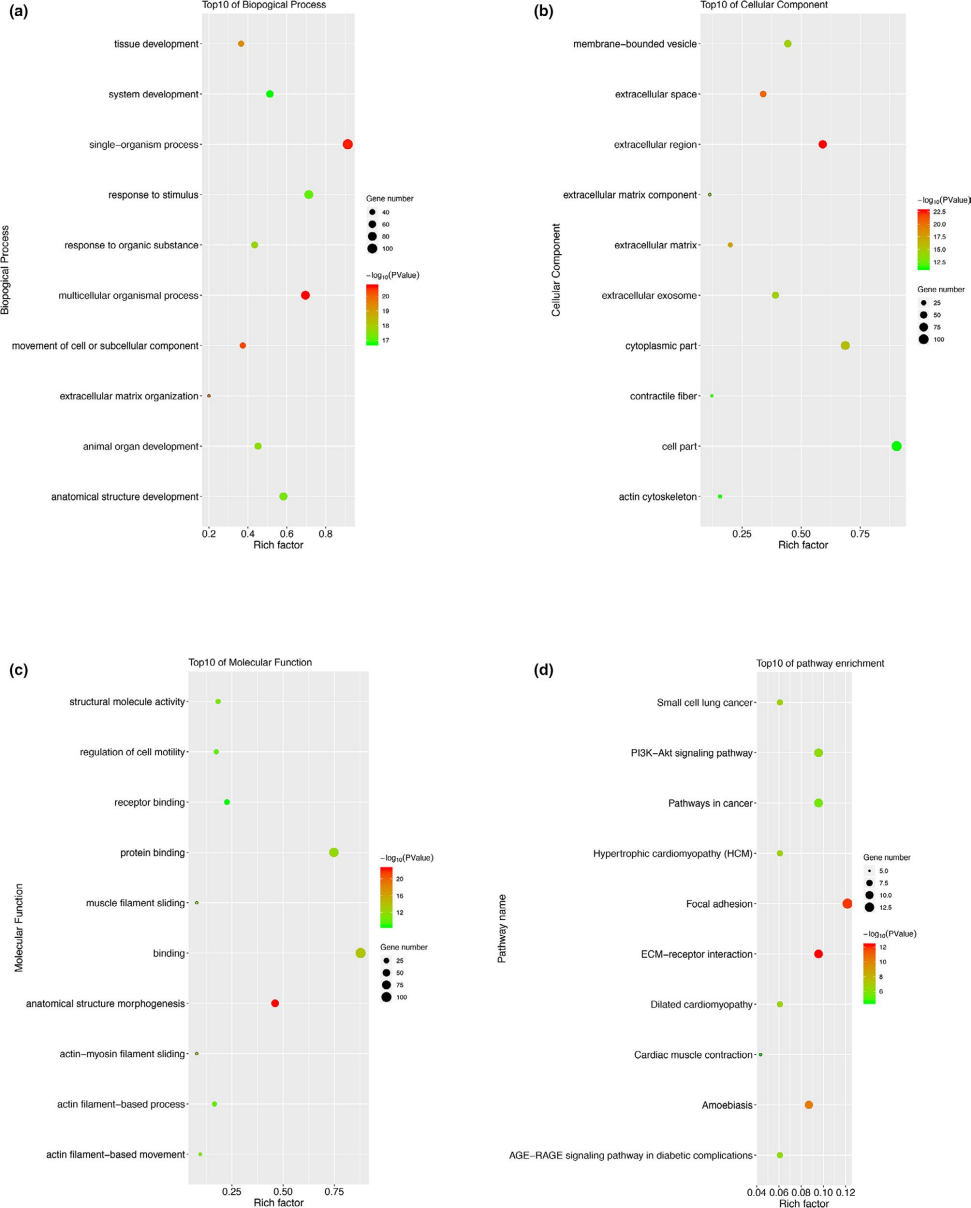
GO analysis and KEGG pathway analysis of the overlapping differentially expressed genes. (a) Top10 of biological process. (b) Top10 of cellular component. (c) Top10 of molecular function. (d) Top10 of KEGG pathway enrichment

To gain a more in‐depth understanding of the common DEGs from three datasets, GO analyses and KEGG pathway enrichment analyses were performed in KOBAS. The top 10 biological processes that these DEGs involved in is presented in Figure 2a, among which response to stimulus and extracellular matrix organization are closely associated with cancer progression. Regarding cellular component, GO analysis results showed that the overlapping DEGs were mainly enriched in extracellular regions, such as extracellular space, extracellular matrix, and extracellular exosome (Figure 2b). It was well‐established that components in the extracellular region may participate in the malignant progression of cancer, and the results highlighted the importance of DEGs in HNSCC progression. For molecular function classification, the DEGs were significantly enriched in the following functions: structural molecule activity, regulation of cell motility, receptor and protein binding, and actin‐myosin filament sliding. (Figure 2c). The results from KEGG analysis showed that among the pathways in which these genes were particularly enriched, many were closely related to cancer progression, such as the PI3K‐Akt signaling pathway, focal adhesion, and ECM‐receptor interaction (Figure 2d). The above findings consistently indicated that these DEGs may modulate HNSCC proliferation and metastasis through multiple signaling pathways.

### 3.3 PPI network of common DEGs and hub gene identification

**Figure 3:**
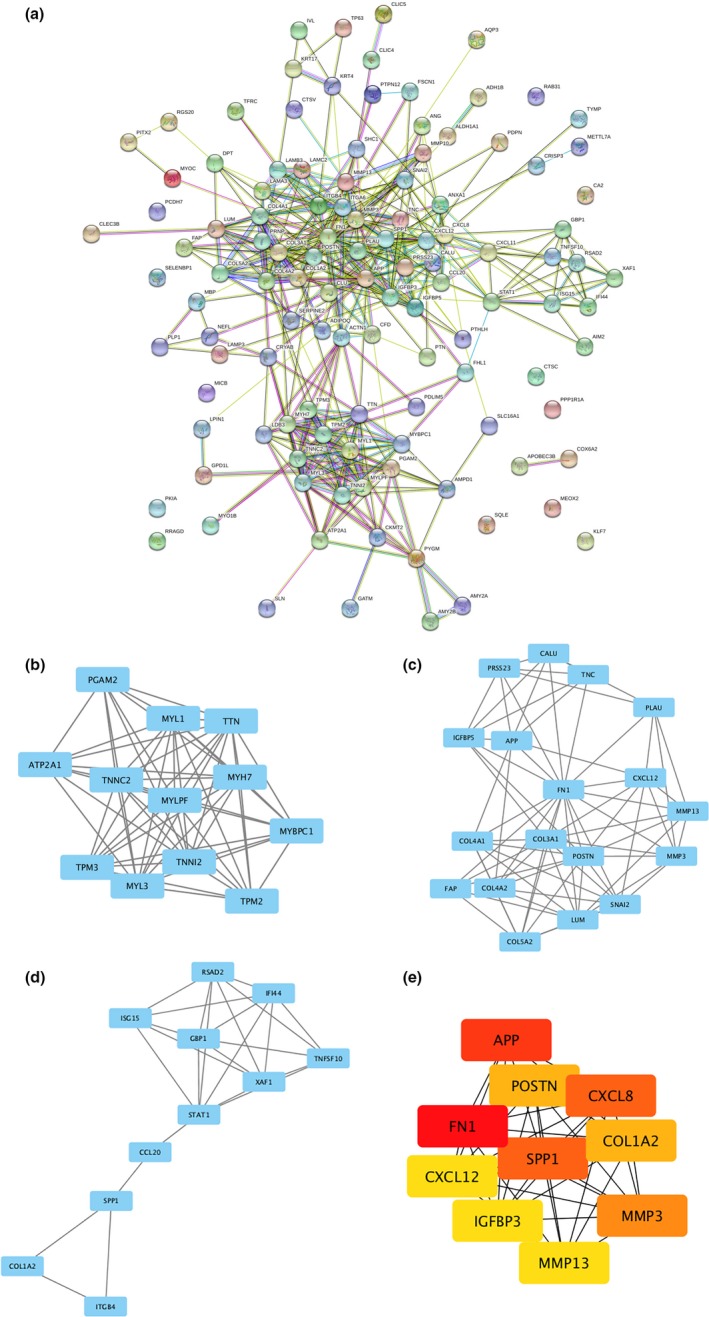
Protein–protein interactions (PPI) network, module analysis, and hub gene identification. (a) PPI network of overlapping differentially expressed genes was constructed in STRING database. (b‐d) Top three modules screened using Molecular Complex Detection (MCODE) in Cytoscape software. (e) Top ten hub genes selected by the CytoHubba in Cytoscape based on the degree of each protein node

**Figure 4:**
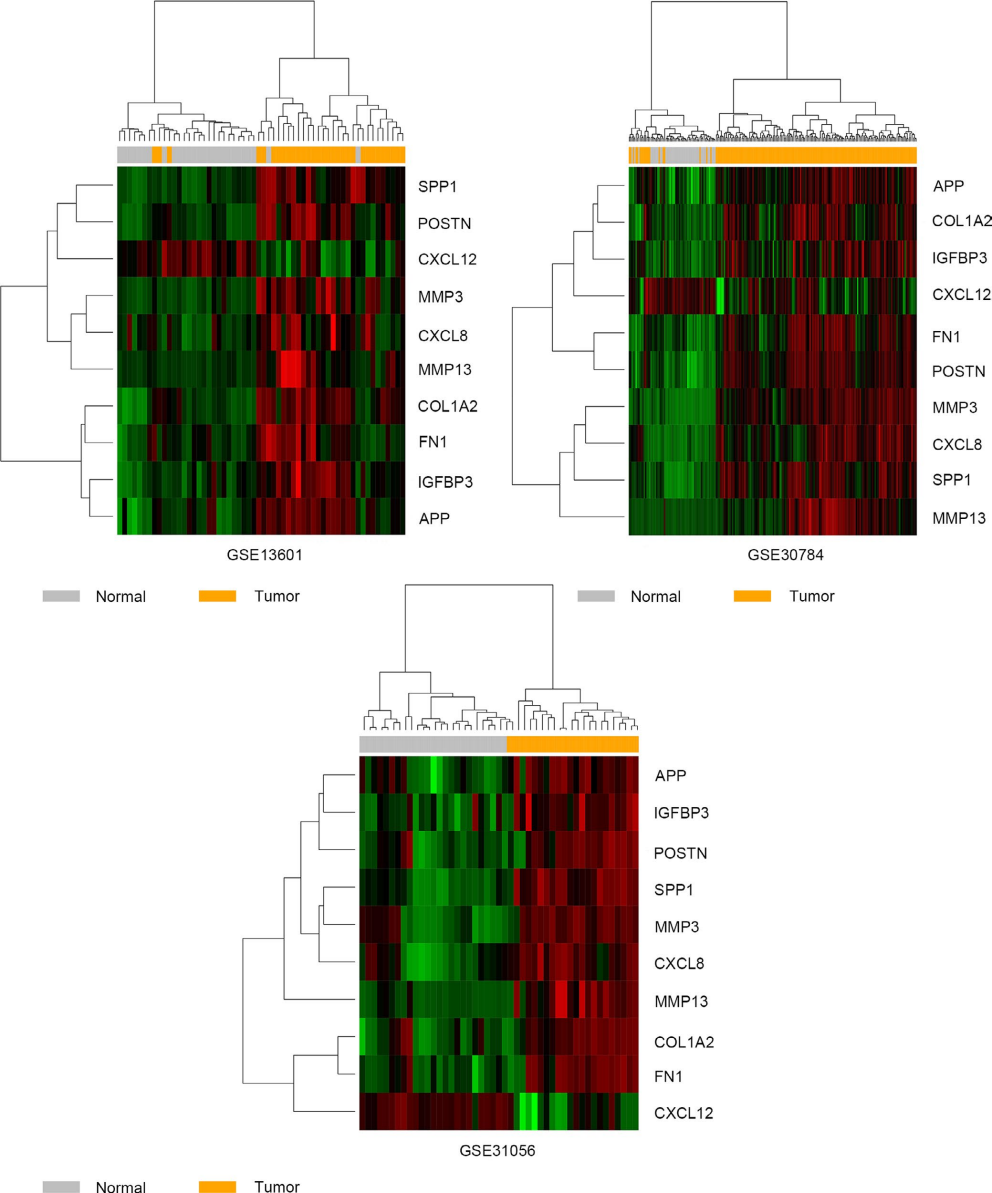
The heatmaps of the overlapping differentially expressed genes profiles in HNSCC and compared normal tissues in GSE13601, GSE30784, and GSE31056. Red indicates upregulation and green indicates downregulation

To further explore the underlying association between DEGs, a PPI network was constructed by using the STRING database (Figure 3a). Then, the top three modules inside the PPI network were identified with the MCODE application in Cytoscape (Figure 3b‐d). For details, module 1 consisted of 12 nodes and 60 edges with the highest degree of 10.909. For module 2, 18 nodes and 75 edges existed, while 11 nodes and 25 edges constituted module 3. Furthermore, cytoHubba was applied to screen out hub genes, and the top 10 genes with the highest degree of connectivity were selected (Figure 3e). The results showed that fibronectin‐1 (FN1, OMIM:135600) was the most outstanding gene with connectivity degree = 40, followed by amyloid precursor protein (APP, OMIM:104760; degree = 26), interleukin‐8 (CXCL8, OMIM:146930; degree = 23), osteopontin (SPP1, OMIM: 166490; degree = 23), stromelysin‐1 (MMP3, OMIM:185250; degree = 20), periostin (POSTN, OMIM:608777; degree = 19), collagen alpha‐2 (COL1A2, OMIM:120160; degree = 19), collagenase 3 (MMP13, OMIM: 600108; degree = 18), insulin‐like growth factor‐binding protein 3 (IGFBP3, OMIM:146732; degree = 18), and stromal cell‐derived factor 1 (CXCL12, OMIM:600835; degree = 18). Then, the heatmaps of hub gene expression in three GEO datasets were plotted as shown in Figure 4. Red indicates upregulation and green indicates downregulation.

### 3.4 Validation of hub gene expression levels in multiple databases

**Figure 5:**
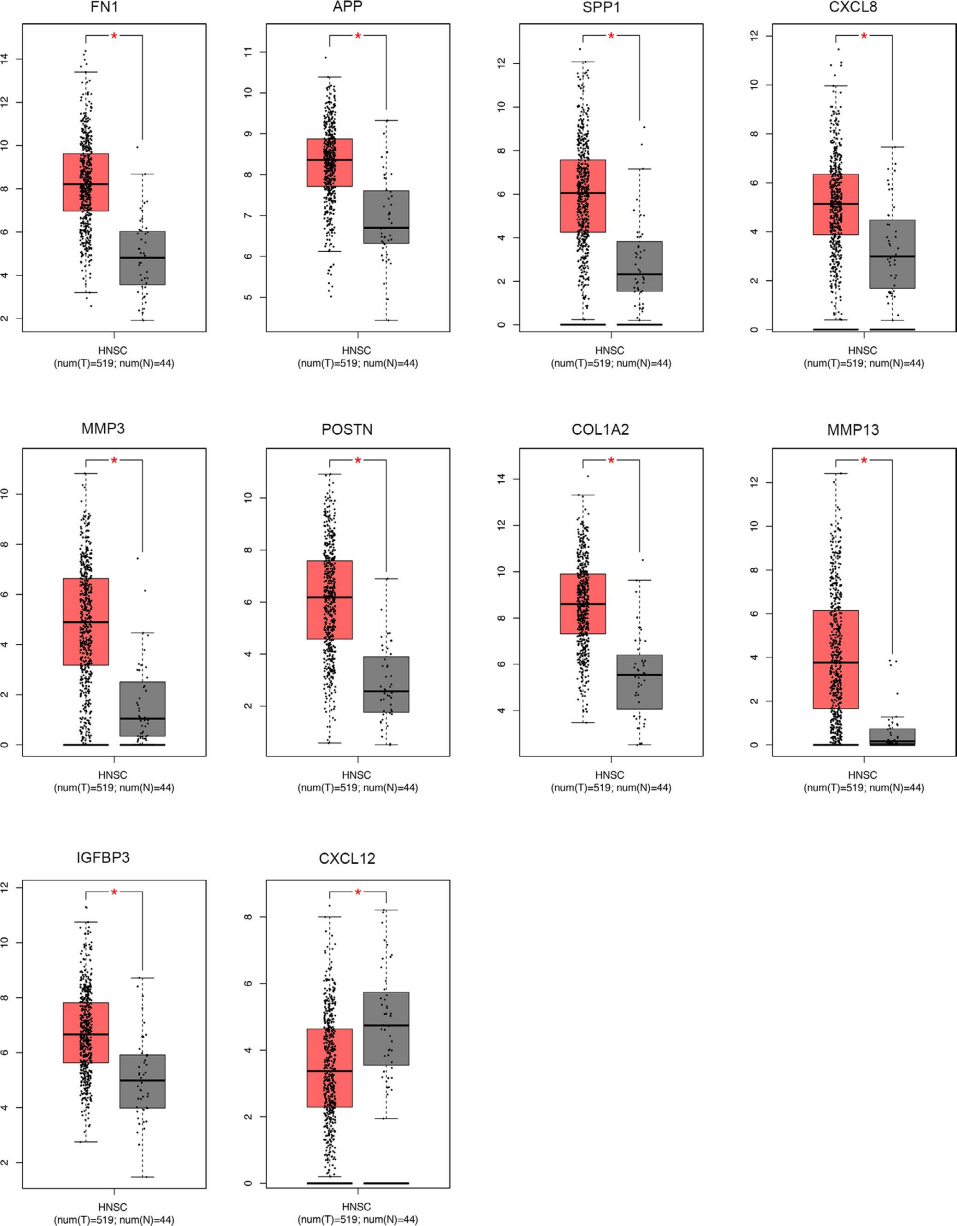
Relative expression of hub genes in HNSCC samples in GEPIA database. The threshold was set as |Log_2_FC|=1, *p* = .05, *p < .05

**Figure 6:**
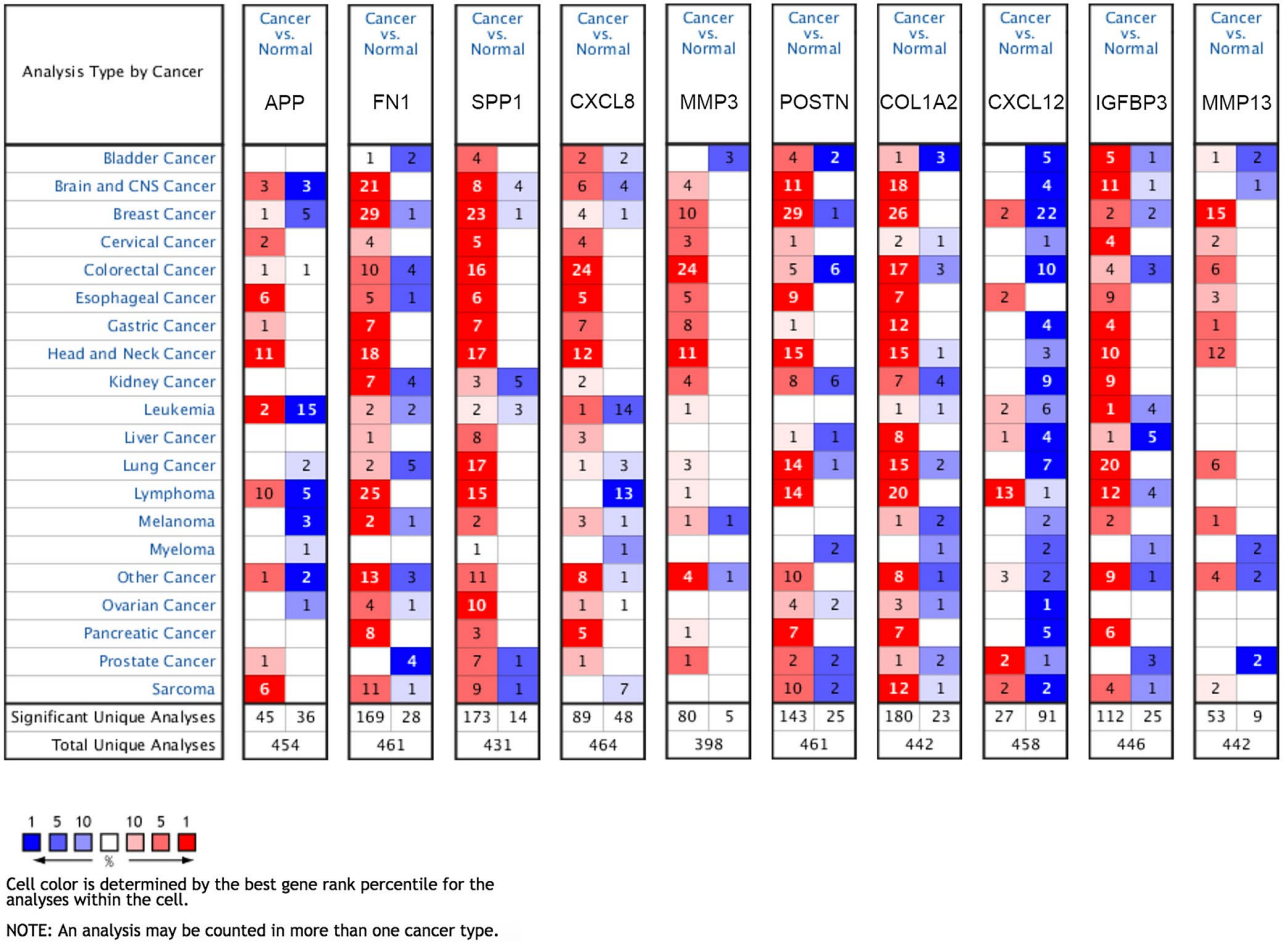
An overview of mRNA levels of hub genes in a variety of cancers based on Oncomine. The transcription levels of hub genes in different types of cancer in Oncomine. The numbers in colored cells show the quantities of datasets with statistically significant mRNA overexpression (red) or underexpression (blue) of target genes. Cell color was determined by the best gene rank percentile for the analysis within the cells. The threshold was set as gene rank percentile (All), *p*‐value (.05), and fold change (1.5)

**Figure 7:**
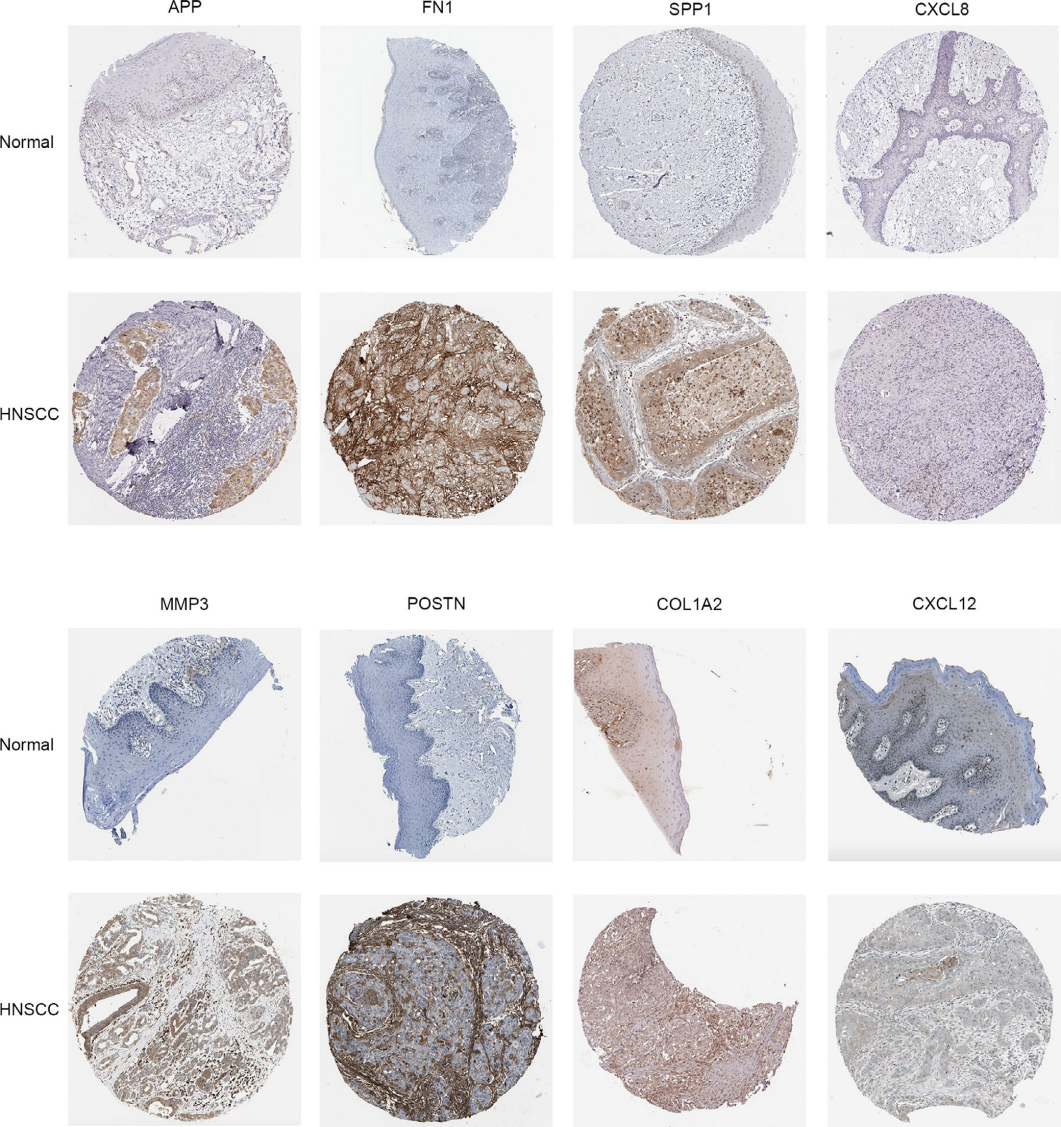
The protein expression of hub genes was manifested in HPA database using immunohistochemistry analysis

We further determined the transcriptional expression differences of hub genes between HNSCC tissues and normal tissues in a variety of datasets in Oncomine and GEPIA. As shown in Figure 5, mRNA levels of hub genes were mostly significantly upregulated in HNSCC samples, with only CXCL12 being downregulated in HNSCC. Furthermore, an overview of hub genes in various kinds of tumors showed that hub genes, except CXCL12, were remarkably overexpressed in HNSCC (Figure 6). Apart from investigating the mRNA level of hub genes, the protein level was also investigated through the HPA database. It can be easily concluded from Figure 7 that the protein expression of hub genes was consistent with mRNA expression, with the majority of genes being overexpressed in HNSCC tissues. Since the immunohistochemistry information about IGFBP3 and MMP13 was not provided in HPA, we have shown eight pairs of staining results in the figure. These findings indicate that the evident differential expression of these hub genes in HNSCC may imply the essential roles of these genes in cancer development and progression.

### 3.5 Survival analysis of hub gene expression in patients with HNSCC

**Figure 8:**
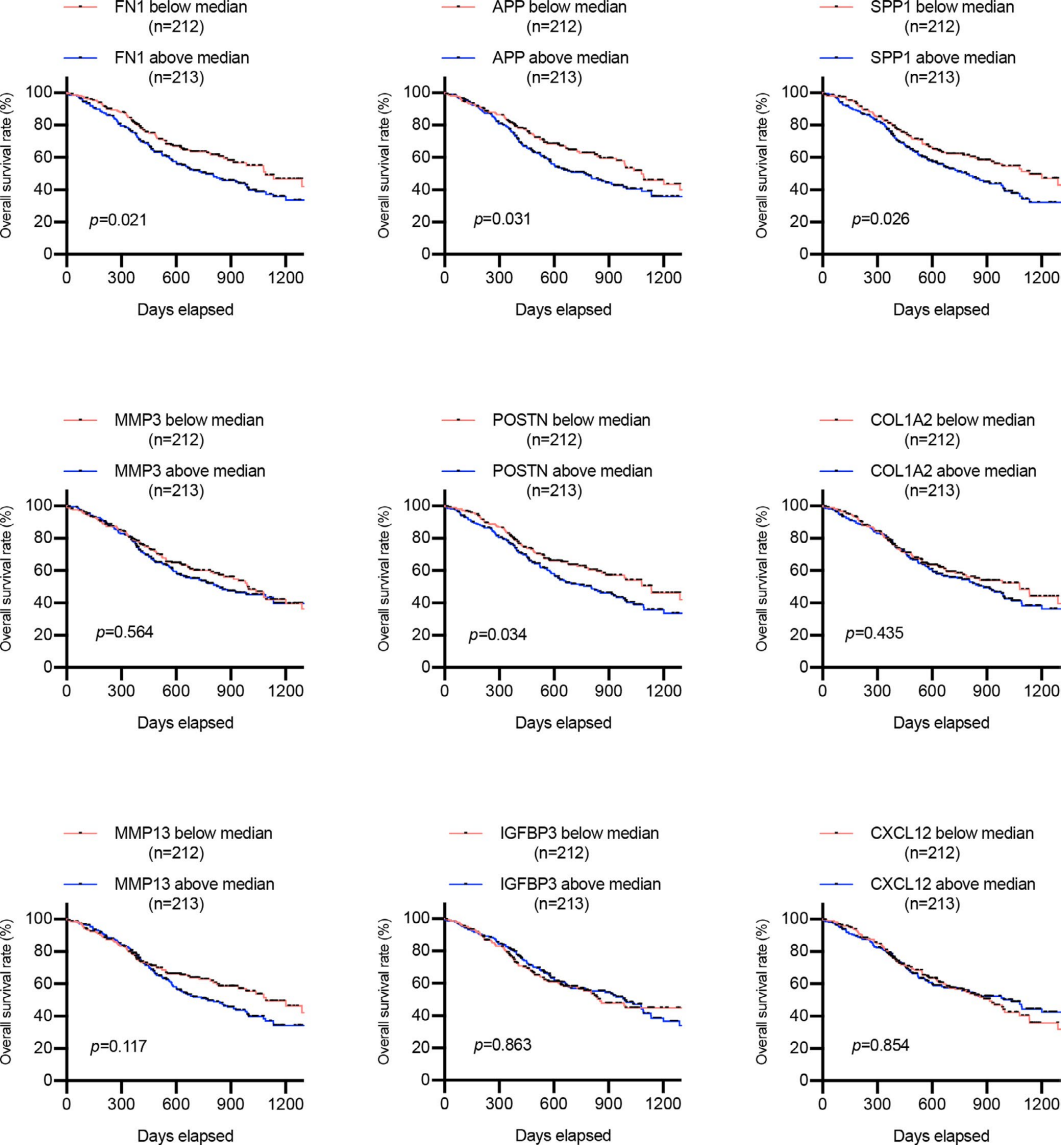
The prognostic value of hub genes in the overall survival of HNSCC patients based on TCGA data

**Figure 9:**
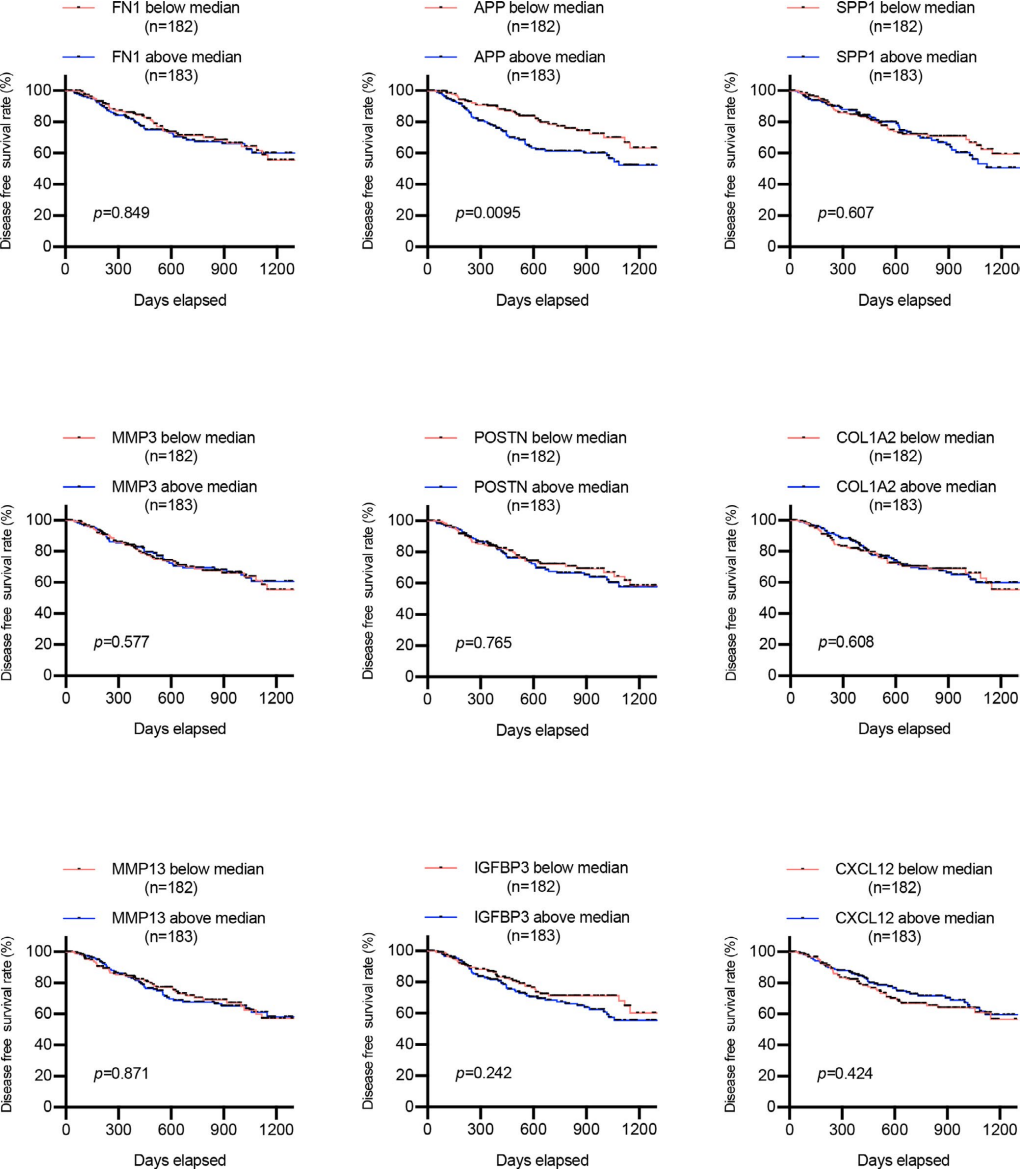
The relationship of hub genes with the disease‐free survival of HNSCC patients based on TCGA data

To further investigate the prognostic values of hub genes in HNSCC patients, we conducted a survival assay based on the TCGA data downloaded from the UCSC Xena database. As suggested in Figure 8, the relative higher expression of FN1, APP, SPP1, and POSTN was associated with poor prognosis of HNSCC patients, while the other genes had no statistical influence on patients’ overall survival. Furthermore, we also detected whether these genes were related to the disease‐free survival of HNSCC patients, and survival curves illustrated that only APP notably affected the disease‐free survival time of HNSCC patients (Figure 9). Evidently, patients with a lower level of APP had better disease‐free survival compared to patients with higher APP expression. From the analysis above, we concluded that APP may be closely correlated with HNSCC overall and disease‐free survival, implying the essential roles that APP may play in HNSCC progression.

### 3.6 Validation of APP and COL1A2 expression in HNSCC clinical samples

**Figure 10:**
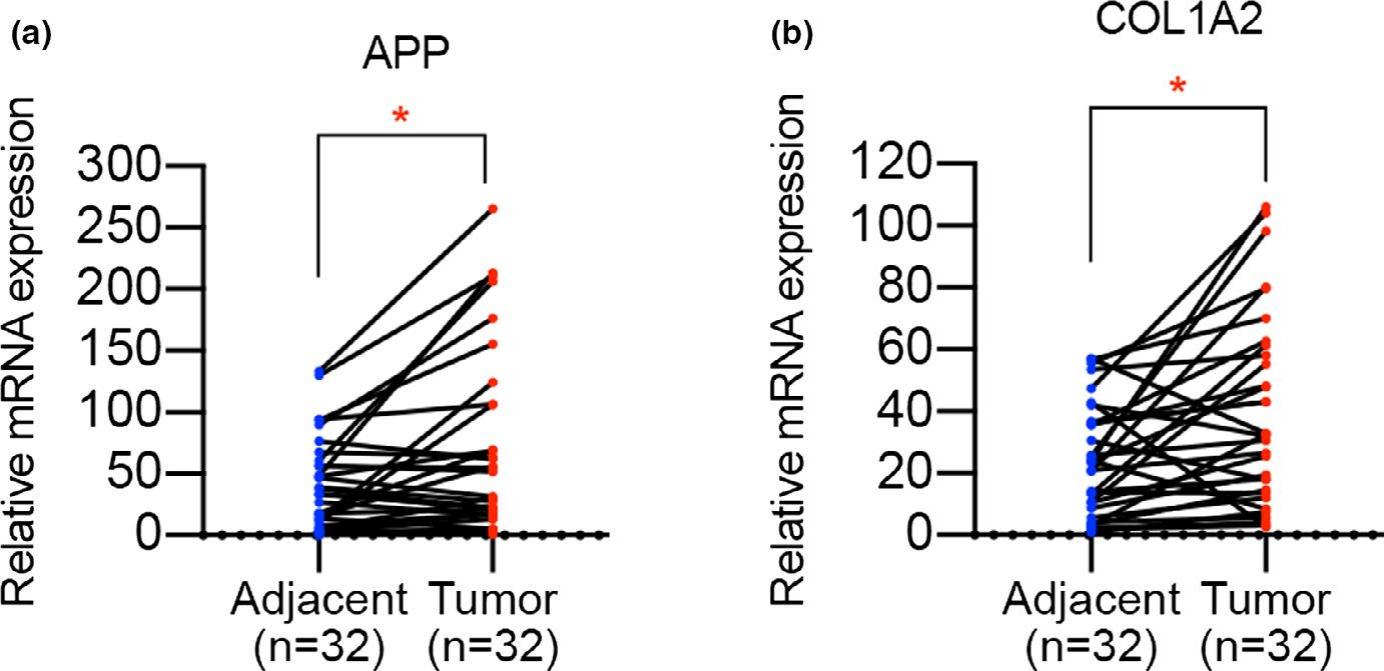
Validation of APP and COL1A2 mRNA expression level in HNSCC samples. (a) APP was upregulated in HNSCC tissues when compared to adjacent matched tissues. (b) The level of COL1A2 was significantly higher in HNSCC samples when compared to adjacent matched tissues. *p < .05

Since APP and COL1A2 from the hub genes have not been investigated before, we determined to further validate the expression level of these two genes in HNSCC samples. The results of qPCR experiment performed in HNSCC tissues suggested that APP and COL1A2 were significantly upregulated in HNSCC tissues when compared to adjacent matched tissues (Figure 10a,b), implying the potential tumor promoter role of them in HNSCC progression.

## 4 DISCUSSION

Despite significant advances in HNSCC treatment protocols, including surgery, radiotherapy, cytotoxic, and novel targeted agents, HNSCC has remained an intractable cancer over the past several decades (Vermorken et al., 2008). Therefore, uncovering the etiological and molecular mechanisms underlying HNSCC is of vital importance for cancer therapy and prevention. For many years, bioinformatics analysis has been playing crucial roles in cancer study, and it facilitates the understanding of carcinogenesis by integrating data at the genome level with systematic bioinformatics methods. Among the multiple bioinformatics strategies, DNA microarray gene expression profiling has been widely applied to explore DEGs involved in tumor genesis, diagnosis, and therapeutic approaches (Fan et al., 2010).

In this study, we first screened DEGs from three GEO datasets and implemented GO/KEGG pathway enrichment analysis. A PPI network was then constructed in the STRING database and the top ten hub genes were selected in Cytoscape. The relative expression of these genes was detected in GEPIA, Oncomine, and HPA databases, suggesting that all the hub genes were dysregulated in HNSCC with statistical significance. Therefore, these hub genes (SPP1, POSTN, COL1A2, FN1, IGFBP3, APP, MMP3, MMP13, CXCL8, and CXCL12) could be utilized as potential diagnostic indicators for HNSCC.

It was demonstrated that overexpression of SPP1 was closely associated with tumor invasion, metastasis and low survival in colorectal cancer (Xu et al., 2017). Additionally, the regulation of SPP1 expression influenced ovarian cancer both in vitro and in vivo, and this process may be related to the β1/FAK/AKT pathway (Zeng, Zhou, Wu, & Xiong, 2018). POSTN, originally isolated as an osteoblast‐specific factor, was illustrated to be involved in the interaction between fibroblasts and cancer cells. It was suggested that the level of stromal POSTN could be induced by TGF‐β3, leading to accelerated growth, migration and invasion of cancer cells (Qin et al., 2016). Another study suggested that POSTN could regulate essential aspects of tumor biology, including proliferation, invasion, matrix remodeling, and dissemination to premetastatic niches in distant organs (Gonzalez‐Gonzalez & Alonso, 2018).

Recently, multiple collagen family members were hypothesized to be involved in carcinogenesis, among which COL1A2 expression was illustrated to be positively related to tumor size and depth of invasion in gastric cancer (Li, Ding, & Li, 2016). Additionally, research on pancreatic cancer suggested that COL1A2 might play a significant role in proliferation, migration, invasion and in vivo xenograft progression, and this process may be mediated through interactions with microRNA (miRNA; Wu et al., 2019). FN1, a glycoprotein component of the extracellular matrix, has the ability of mediating interactions between cells and the extracellular matrix and plays essential roles in cell adhesion, migration, growth, and differentiation (Zhou, Shu, & Huang, 2019). For thyroid cancer, FN1 upregulation acts as an important determinant of cancer aggressiveness and participates in the epithelial‐to‐mesenchymal transition (Sponziello et al., 2016). Moreover, FN1 was found to be able to avoid the apoptotic activities of ovarian cancer cells caused by therapeutic agents, implying that the regulation of FN1 may improve the therapeutic effect (Xing et al., 2008).

As an N‐linked glycosylated, phosphorylated and secreted protein, IGFBP3 has been demonstrated to inhibit cell proliferation and induce cell apoptosis of multiple common cancers, including prostate, breast, lung, and colorectal cancers (Gallagher & LeRoith, 2010; Renehan et al., 2004; Yu & Rohan, 2000). However, IGFBP3 exerted tumor‐promoting roles in other cancers and was associated with poor prognosis of patients (Bao et al., 2016). From our perspective, the opposite effect of IGFBP3 may be dependent on the different tumor origin and cell context and the overexpression of IGFBP3 in HNSCC implied that it may function as a tumor promoter. APP was hypothesized to be a promoting factor for a variety of malignant tumors. The soluble secreted form of APP (sAPP), from its N‐terminal domain was shown to exhibit a growth factor‐like function (Rossjohn et al., 1999), and the proteolytic products of APP processing have growth‐promoting qualities and regulate cell cycle in multiple cancers (Hansel et al., 2003; Meng, Kataoka, Itoh, & Koono, 2001). Another study also suggested that the tumor promoter role of APP may be obtained by modulating the expression of metalloproteinase and EMT‐related genes (Miyazaki, Ikeda, Horie‐Inoue, & Inoue, 2014).

Matrix metalloproteinases (MMPs), which are well‐known to have the ability to degrade the extracellular matrix, were suggested as strong mediators of cancer invasion and metastasis. By releasing growth factors from the extracellular matrix, MMPs can regulate cancer cell growth, apoptosis and angiogenesis. Increased expression of MMPs was indicated to result in poor clinical outcome in several cancer types, emphasizing the role of MMPs in cancer progression (Brinckerhoff & Matrisian, 2002; Coussens, Fingleton, & Matrisian, 2002; Egeblad & Werb, 2002). In this study, MMP3 and MMP13 from MMPs were screened out as DEGs in HNSCC, implying that they may play some roles in HNSCC progression. A previous study showed that MMP3 exhibited increased expression in various kinds of tumors and influenced cancer initiation and neoplastic risk (Radisky et al., 2005; Sternlicht, Bissell, & Werb, 2000. In vivo experiments consistently demonstrated that the silencing of MMP3 expression led to reduced tumor growth and metastatic potential of melanoma cells (Shoshan et al., 2016). Moreover, it was demonstrated that the overexpression of MMP13 in esophageal squamous cell carcinoma could promote cancer cell aggressiveness (Osako et al., 2016). Also, MMP13 regulation was demonstrated to be the cause of RKIP‐mediated inhibition of local cancer invasion (Datar et al., 2015).

Under normal conditions, chemokine networks regulate a good number of cellular, physiological, and immune processes. However, these functions can be appropriated by cancer cells to facilitate growth, proliferation, and metastasis (Sleightholm et al., 2017). CXCL8 has been determined to be involved in a multiple of cancers, such as breast cancer, prostate cancer, lung cancer, and melanoma. However, the detailed mechanisms underlying CXCL8‐mediated cancer progression may be diverse. For example, in breast cancer, CXCL8 could facilitate tumorigenesis by modifying the microenvironment. For prostate cancer, CXCL8 upregulates the expression of CXCR7, which could interact with EGFR directly to induce cancer cell growth (Singh & Lokeshwar, 2011). In melanoma, the metastatic potential of CXCL8 depends on its ability to promote vascularization, activate MMP2, and enhance anoikis resistance (Luca et al., 1997). The CXCL12/CXCR4 system is overexpressed in a large variety of tumors, and this axis has been increasingly identified as an important target in cancer growth, metastasis, relapse, and resistance to therapy (Albert et al., 2013). CXCL12, in conjunction with its receptor, has been reported to promote pancreatic cancer development, invasion, and metastasis through the management of the tumor microenvironment via a complex crosstalk with other pathways (Sleightholm et al., 2017). Overall, the above conclusions establish a foundation for our findings that hub genes may act as efficient biomarkers and potential therapy targets in HNSCC.

Moreover, survival analysis results suggested that FN1, APP, SPP1, and POSTN may be prognostic indicators in HNSCC patients. Furthermore, given that APP was the only gene whose expression level was significantly associated with the disease‐free survival of HNSCC patients, this gene may be involved in cancer progression. Further comprehensive and in‐depth investigation of these genes would be highly valuable.

Finally, based on the validation of APP and COL1A2 upregulation in HNSCC tissues, we suggested that APP and COL1A2 may play significant roles in HNSCC progression. Further in‐depth investigation regarding the biological effect of APP and COL1A2 on HNSCC cells remains to be implemented.

## 5 CONCLUSION

Based on integrated bioinformatics analysis including GO and KEGG pathway enrichment, PPI network, module analysis and hub gene identification, the present study investigated DEGs that may have the potential to serve as reliable molecular biomarkers for the diagnosis and prognosis of HNSCC. Moreover, APP and COL1A2 were validated to be upregulated in HNSCC samples. Further research is merited to explore the biological functions of these genes and the underlying mechanisms involved in the pathogenesis of HNSCC.

## CONFLICT OF INTEREST

None declared.
